# A retrospective study of infections after primary VP shunt placement in the newborn with Myelomeningocele without prophylactic antibiotics

**DOI:** 10.1186/1743-8454-7-S1-S43

**Published:** 2010-12-15

**Authors:** Dorte Clemmensen, Mikkel Mylius Rasmussen, Claus Mosdal

**Affiliations:** 1 Department of Neurosurgery, Aarhus University Hospital, Canada

## Background

We aim to correlate the frequency of infections after VP shunt placement in neonates with myelomeningocele (MMC) who did not receive prophylactic antibiotics, to the timing of VP shunt placement and the frequency of Cerebrospinal fluid (CSF)leakage at the MMC wound.

## Materials and methods

59 newborns with MMC underwent VP shunt insertion in the period 1983-2007. We reviewed retrospectively all records.

## Results

The relative risk (RR (95%)) of having an infection is significant higher RR=4,69 (1.145397 -19.23568) (P = .03761817)and neuroinfection showed a tendency towards : RR = 3.5 (.7067445 - 17.03112) (P = .15414095) in newborns without symptomatic Hydrocephalus at birth when we had a wait and watch policy (late shunt placement) compared to newborns with prompt shunt placement. The relative risk (RR 95%) of having a infection was highly significant: RR = 6,8 (3.314154 -13.95228) (P= 1.235e-07) and also neuroinfections RR = 4,76 (2.043019 - 11.09025) ( P = .00044478) if the child presented MMC wound CSF leakage before VP shunt insertion.

## Conclusions

Centres with a conservative antibiotic policy should be even more careful to avoid CSF leakage before shunt placement as this gives a highly significant increased risk of both infections in total and neuroinfections and they should reconsider this conservative policy in newborns with MMC due to the significant high infections rate.

